# Prolonged Postoperative Vasoplegia in Pediatric Patients on Chronic Angiotensin II Blocker Treatment

**DOI:** 10.3389/fcvm.2018.00121

**Published:** 2018-09-04

**Authors:** Nischal R. Pandya, Nelson Alphonso, Quyen Tu, Prem Venugopal, Luregn J. Schlapbach

**Affiliations:** ^1^Pediatric Cardiac Surgery, Lady Cilento Children's Hospital, Brisbane, QLD, Australia; ^2^Mater Research Institute, The University of Queensland, Brisbane, QLD, Australia; ^3^Pediatric Critical Care Research Group, Mater Research Institute, The University of Queensland, Brisbane, QLD, Australia; ^4^Pediatric Intensive Care Unit, Lady Cilento Children's Hospital, Brisbane, QLD, Australia; ^5^Faculty of Medicine, The University of Queensland, Brisbane, QLD, Australia

**Keywords:** angiotensin blocker, pediatric, antihypertensive, cardiac surgery, vasoplegia

## Abstract

Prolonged postoperative vasoplegia is known to occur following cardiac surgery in patients on chronic angiotensin II receptor blocker (ARB) treatment in adults. The perioperative management of these drugs in the pediatric population is not well described and here we would like to highlight this fact. While ARBs are increasingly used in children and adolescents with hypertension, there is lack of data to guide optimal pre-surgical management in the pediatric age group. We report two cases of prolonged vasoplegia following cardiopulmonary bypass occurring in adolescent patients on chronic ARB therapy and the importance of cessation of these drugs preoperatively.

## Introduction

Angiotensin II receptor blockers (ARBs) are commonly used antihypertensive drugs in adults. They selectively block the binding of angiotensin II to the angiotensin II type 1 subtype receptor (AT1), which is found in the cardiovascular and renal systems. Prolonged vasodilatory effects after cardiac surgery have been described in adults on chronic ARB treatment and where cessation of ARBs up to 24 h prior to surgery is recommended (1). While ARBs are increasingly used in children and adolescents with hypertension, there is a lack of data to guide optimal pre-surgical management in the pediatric age group. Angiotensin receptor blockers as monotherapy or as fixed-dose combinations contributed to 26.3% of pediatric hypertension prescriptions in Europe (2).

We report two cases of sustained vasoplegia following cardio-pulmonary bypass in adolescent patients treated with regular long-term ARBs (candesartan cilexetil) preoperatively. The Health Service Human Research Ethics Committee endorsed this work and written parental permission of both patients was granted for this case report.

## Case presentations

### Case 1

A 17-year-old male with Marfan's syndrome and a family history of Marfan's and aortic dissection in one parent, was diagnosed with a dilated aortic root (sinuses of Valsalva 47 mm) with trivial aortic regurgitation. He took candesartan 8 mg every morning including the day of surgery. He underwent a valve-sparing aortic root replacement. Maintenance of normotension during cardiopulmonary bypass required the administration of noradrenaline. Postoperatively he developed severe vasoplegia with a rise in serum lactate to 8.1 mmol/L that responded to treatment with noradrenaline. He did not have any other organ dysfunction and was extubated after 12 h. He required vasoconstrictor therapy for 53 h. He made an uneventful recovery thereafter and was discharged home on postoperative day 8.

### Case 2

A 14-year-old male with Marfan's syndrome and progressive aortic root dilatation (sinuses of Valsalva 47 mm), moderate mitral regurgitation and significant pectus carinatum, underwent a valve sparing aortic root replacement, aortic valve repair, mitral valve repair and concomitant Ravitch procedure. He took candesartan 4 mg twice a day including the night prior to surgery. Maintenance of normotension during cardiopulmonary bypass required the administration of noradrenaline. Shortly after transfer to the intensive care unit he developed profound hypotension despite fluid boluses and increasing vasopressor doses and required a brief period (2 min) of cardiopulmonary resuscitation. His inotrope requirement included adrenaline up to 0.1 mcg/kg/min, noradrenaline up to 0.08 mcg/kg/min, dopamine up to 10 mcg/kg/min, and vasopressin up to 0.6mU/kg/min. His serum lactate postoperatively increased to 9.7 mmol/l before normalizing over 24 h. There was no evidence of other organ dysfunction. He required vasoconstrictor therapy for 106 h and mechanical ventilation for 72 h after which time he made an uneventful recovery. The clinical parameters and the vasoactive medications used have been summarized in Table 1.

**Table 1:** Summarizes the clinical parameters of the cases including the vasoactive medications used and the vasoactive inotropic score (VIS).

**PARAMETERS**	**CASE 1**		**CASE 2**	
**Blood pressure (mm Hg)**	**Pre op**	**Post op**	**Pre op**	**Post op**
Systolic	102	75	94	67
Diastolic	62	31	51	32
Mean	75	46	65	44
Cardiopulmonary bypass time (minutes)	179		230	
Cross clamp time (minutes)	154		204	
Fluid bolus	1.1L fluid in the form of albumin 4% was infused over an hour		970 ml fluid in the form of albumin 4%, blood products were infused over an hour	
Vasoactive support				
Vasopressin (mU/kg/min)	–		0.6	
Adrenaline (μg/kg/min)	–		0.1	
Noradrenaline(μg/kg/min)	0.05		0.08	
Dopamine (μg/kg/min)			10	
Milrinone (μg/kg/min)	0.3			
Vasoactive Inotropic Score	8		34	

Both patients had gas induction for anesthesia and maintenance using Sevoflurane (Case 1: 1.7% and case 2: 2.9%). Both had a combination of antegrade-retrograde intermittent cold blood cardioplegia instituted and the cardiopulmonary bypass was maintained at normothermia. At the end of the procedure both patients underwent modified ultrafiltration and were transferred to the intensive care unit with a positive fluid balance of 140 and 790 ml respectively.

## Discussion

We report on two adolescents with prolonged ARB-induced vasoplegia resulting in substantial early postoperative morbidity. Both patients were receiving regular ARBs preoperatively as prophylaxis for the aortopathy associated with Marfan's syndrome to reduce the risk of aortic aneurysm formation (3). Postoperatively, both patients developed hypotension poorly responsive to fluid boluses and requirement for progressively increasing doses of vasoconstrictors. Both required inotrope administration for >48 h and >72 h stay in PICU, with a Vasoactive-Inotropic Score (VIS) of 8 and 34 respectively.

In contrast, similar patients operated at our institution are usually extubated, weaned off inotropes within the first 4 h postoperatively and discharged to the ward within 12–24 h. Both patients were not on routine pre-operative intravenous heparin. Other causes of severe vasoplegia, including sepsis and over sedation were ruled out in both patients. Our report demonstrates that ARBs can have profound and prolonged effects in pediatric patients undergoing heart surgery that can result in potentially life-threatening vasoplegia.

Circulating human angiotensin II exerts an array of effects including sodium and water reabsorption, which increases intravascular fluid volume leading to increased cardiac preload and stroke volume, systemic arteriolar vasoconstriction leading to increased vascular resistance and cardiac afterload. Candesartan cilexetil, the esterified prodrug of candesartan, is metabolized to candesartan during its intestinal absorption. Pharmacokinetic studies in children have demonstrated a half-life of 5.7–6.7 h (4). The serum levels of ARBs are unlikely to be affected by ultrafiltration (5).

The association between angiotensin converting enzyme inhibitors (ACEI) and post-operative hypotension was first recognized more than two decades ago in adults (6, 7). In a previous study Ajuba-Iwuji et al reported that preoperative ACE inhibitor and ARB use in pediatric patients undergoing cardiac surgery did not significantly increase the incidence of hypotension after induction of anesthesia and did not significantly increase vasoconstrictor requirements upon weaning from CPB; however, patients on ACE inhibitor/ARB therapy tended to have a higher VIS than patients in the control group (8). This study has important limitations acknowledged by the authors including small and discrepant sample size (18 vs. 132), unmatched groups of patients, different mean age (6.4 ± 6.1 years vs. 4.3 ± 5.5 years), unmatched diagnosis, potential of under reporting and recording bias and combining patients taking ACEI and ARB into one group (both these drugs act at different levels). The analysis time-point was terminated at 90 min even though the VIS score showed a higher trend in the group of patients taking ACEI and ARBs preoperatively (11.3 vs. 6.7). The difference was not statistically significant which is likely a function of the study being underpowered to detect a true difference. However, in a more recent study, withholding ACEI/ARBs before major non-cardiac surgery was associated with a lower risk of death and postoperative vascular events in adults (9).

ACEI and ARBs are being increasingly used in adolescents, thus exposing them to the risk of severe vasoplegia in the perioperative period (10). Treatment of refractory vasoplegia in shocked patients can be challenging, and may require the use of non-adrenergic vasoconstrictors like vasopressin or angiotensin II (11). We believe that more emphasis should be focused on prevention through parental advice with clear instructions to stop ARBs at least 24 h prior to elective cardiac surgery in children like the recommendation in adults (1).

## Concluding remarks

We report two adolescents with prolonged postoperative vasoplegia after chronic ARB exposure. Timely cessation of drugs targeting the angiotensin II system at least 24 h prior to surgery should be included in standard perioperative protocols and patient information leaflets for pediatric cardiac surgical patients. Further studies in this group of patients with an increasing use of ARB are warranted.

## Ethics statement

The Children's Health Queensland Hospital and Health Service Human Research Ethics Committee notes that consent has been obtained for all parties involved and that this has been conducted within all best practice ethical guidelines and under these circumstances the Committee endorses this work.

## Author contributions

NP and LS were involved in data collection. NP, NA, QT, PV, and LS were involved in manuscript preparation and review.

### Conflict of interest statement

The authors declare that the research was conducted in the absence of any commercial or financial relationships that could be construed as a potential conflict of interest.
